# A Case Series of New-Onset Ulcerative Colitis Following Recent Diagnosis of COVID-19

**DOI:** 10.1097/PG9.0000000000000383

**Published:** 2023-11-27

**Authors:** Matthew D. Swatski, Panamdeep Kaur, Rachel E. Borlack, Shonnell McBain, Joshua Uffer, Osama Almadhoun

**Affiliations:** From the * Division of Gastroenterology and Nutrition, Department of Pediatrics, John R. Oishei Children’s Hospital, State University of New York at Buffalo Jacobs School of Medicine and Biomedical Sciences, Buffalo, NY; † Division of Pediatric Gastroenterology, Department of Pediatrics, Connecticut Children’s Hospital, University of Connecticut School of Medicine, Hartford, CT; ‡ Division of Gastroenterology, Hepatology and Nutrition, Department of Pediatrics, Children’s Hospital at Montefiore, Albert Einstein College of Medicine, Bronx, NY.

**Keywords:** pediatric ulcerative colitis, COVID-19, inflammatory bowel disease (IBD)

## Abstract

There have been only 2 reported cases of new-onset ulcerative colitis in pediatrics following acute coronavirus disease 2019 (COVID-19). We are reporting a case series of 3 adolescent female patients, 2 of whom were vaccinated against COVID-19, who developed new-onset ulcerative colitis following a recent diagnosis of COVID-19 infections at a singular pediatric hospital. This case series should be an impetus to clinicians who have pediatric patients with persistent symptoms of hematochezia, diarrhea, and abdominal pain following acute COVID-19 infection to consider further workup for inflammatory bowel disease.

## INTRODUCTION

This is the third reported case series of 3 pediatric patients with new-onset ulcerative colitis (UC) following acute coronavirus disease 2019 (COVID-19). There have been 2 cases involving a 9-year-old female and a 13-year-old female who developed UC following COVID-19 (1,2). There have been a small number of cases of new-onset inflammatory bowel disease (IBD) following acute COVID-19 in adults including a previously healthy, fully-vaccinated 21-year-old male (3).

## CASES

The cases are summarized in Table 1.

**TABLE 1.** A summary of the 3 patients included in the case series

	Patient 1	Patient 2	Patient 3
Age and sex	16-year-old female	16-year-old female	12-year-old female
COVID-19 diagnosis	3 weeks before the presentation	8 weeks before the presentation	3 weeks before the presentation
COVID-19 course	No hospitalizations or use of antiviral treatments	No hospitalizations or use of antiviral treatments	No hospitalizations or use of antiviral treatments
Presenting symptoms	3 weeks of abdominal pain and hematochezia	1 month of abdominal pain, hematochezia, headaches, nausea and fatigue	Abdominal pain and hematochezia
Family history	Crohn disease in her paternal aunt and paternal second cousin	No family history of IBD or autoimmune disorders	No family history of IBD or autoimmune disorders
Pediatric Ulcerative Colitis Activity Index Score	70 (Severe)	65 (Severe)	70 (Severe)
Normal ranges		Initial lab workup	
12.0–15.0 g/dL	Hgb = 13.8 g/dL	Hgb = 12.8 g/dL	Hgb = 12.4 g/dL
0–20 mm/h	ESR = 10 mm/h	ESR = 6 mm/h	ESR = 18 mm/h
0.20–10.0 mg/L	CRP = 8.12 mg/L	CRP = 1.9 mg/L	CRP = 49.53 mg/L
0–120 mcg/g	Positive fecal occult blood	Stool calprotectin = 2200 µg/g	Stool calprotectin= 1944 µg/g
<50 µg/g Normal 50–120 µg/g Borderline >120 µg/g Elevated	Positive stool lactoferrin	Stool lactoferrin = 450 µg/g	Positive stool lactoferrin
Infectious stool studies*	Negative	Negative	Negative
COVID-19 vaccination status	2 Pfizer-BioNTech COVID-19 vaccines	2 Pfizer-BioNTech COVID-19 vaccines	Unvaccinated
Colonoscopy	Diffuse severe inflammation characterized by adherent blood, altered vascularity, edema, erosions, erythema, and confluent ulcerations was found throughout the colon	Inflammation in a continuous and circumferential pattern from the anus to the cecum	Diffuse moderate colitis with loss of vascularity and mild shallow ulcers found throughout the entire colon
Mayo score	3	3	2
Paris classification	E4, S1	E4, S1	E4, S1
Histopathology	Diffuse chronic active colitis with normal histopathology of terminal ileum, indicative of ulcerative colitis	Chronic active pancolitis with cryptitis from the rectum through the cecum, and normal terminal ileum pathology consistent with a diagnosis of ulcerative colitis	Chronic active pancolitis throughout the colon with normal terminal ileum
Treatment	Oral prednisone 40 mg for outpatient induction, mesalamine, and infliximab biosimilar at 10 mg/kg/dose.	Oral prednisone 40 mg for outpatient induction and mesalamine for maintenance therapy. Due to poor response, therapy was escalated to infliximab originator at 10 mg/kg/dose. 40 days after diagnosis, she first tested positive for *C. Diff.* and oral vancomycin was initiated	Oral prednisone 40 mg daily for outpatient induction therapy. She was subsequently readmitted for worsening hematochezia and initiated on infliximab biosimilar at 10 mg/kg/dose
Response	Symptoms improved after starting induction with infliximab	Symptoms improved after starting infliximab and vancomycin	Symptoms improved after starting induction with infliximab

* Infectious stool studies include *Salmonella*, *Shigella*, *Campylobacter*, *E. coli* 0157:H7, Shiga Toxin 1 and 2, *Yersinia*, *C. difficile*, *Giardia* species and *Cryptosporidium.*

## DISCUSSION

An infectious trigger of IBD has been previously described in intestinal infections such as *Cytomegalovirus*, *Mycobacterium avium* subspecies paratuberculosis, adherent-invasive *Escherichia coli*, and *Campylobacter* species (4). The SARS-CoV-2 virus can presumably trigger IBD via 2 different intracellular pathways, the endosomal pathway and the membrane (cytoplasmic) pathway. This mechanism, as described in Dvornikova et al. (5), is summarized below. In the endosomal pathway, the SARS-CoV-2 S glycoprotein attaches to intestinal enterocytes via the ACE2 receptor that is highly expressed in intestinal enterocytes. The virus is ferried to the endosome. In the endosome, the viral RNA activates the TRIF and the MyD88-dependent pathway pathways, respectively. The activation of the TRIF pathway eventually leads to the induction of IRF3 which produces IFN-α,β. The MyD88 pathway leads to the production of cytokines such as IL-1, IL-6, and TNF-α. Another membrane receptor, TLR4 also attaches to the SARS-CoV-2 S glycoprotein. TLR4 also induces IRF3. The SARS-CoV-2 Viroporin 3a protein via IL-1β and the recognition of viral RNA and proteins leads to the assembly of the NLRP3- inflammasome that has been implicated in worsening colonic mucosal lesions in patients with IBD. The adaptive immune response also plays a role in which the macrophages and dendritic cells display the SARS-CoV-2 virus to T-cells that differentiate into Th1 and Th17 cells. Th1 and Th17 cells then produce a cytokine storm, as described in Wu et al. (6), that decreases the SARS-CoV-2 load but precipitates the development of IBD.

Our case series suggests that COVID-19 vaccination does not prevent the development of UC, but larger sample sizes would be needed. It is also notable that all 3 patients were responsive to infliximab, which may further investigation and may lead to insights into how the virus can trigger IBD. There are only a few reported cases of adult patients who developed IBD following COVID-19 and 2 case reports of pediatric patients who developed new-onset Crohn disease, including 1 with concurrent multi-inflammatory syndrome in children (7). Finally, this case series confirms epidemiological assessments of IBD in New York City that have suggested a link between COVID-19 and UC (8).

## CONCLUSION

In a genetically predisposed individual, any viral infection can trigger the onset of IBD. There are some unique pathogenetic pathways that could trigger the onset of IBD after COVID-19 infection and it is important to note that in this small case series, the vaccination did not prevent the dysregulation of immune pathways resulting in IBD. However, larger studies are needed to determine if the risk of developing IBD is higher with COVID-19 as compared to other infections. We hope to impress upon our clinical colleagues the importance of considering new-onset IBD in patients with persistent hematochezia, diarrhea, and abdominal pain following acute COVID-19.

## ACKNOWLEDGMENTS

The respective legal guardians of all 3 patients provided verbal consent to their inclusion in this case series.
